# Iron deficiency anemia and the risk of new-onset tinnitus in female patients: a cohort study

**DOI:** 10.3389/fnut.2025.1704946

**Published:** 2025-11-28

**Authors:** Kuo-Chuan Hung, Hsiu-Lan Weng, Yi-Chen Lai, Yao-Tsung Lin, Jheng-Yan Wu, Chien-Hung Lin, I-Wen Chen

**Affiliations:** 1 Department of Anesthesiology, Chi Mei Medical Center, Tainan City, Taiwan; 2 Department of Anesthesiology, E-Da Hospital, I-Shou University, Kaohsiung City, Taiwan; 3 Department of Nutrition, Chi Mei Medical Center, Tainan City, Taiwan; 4 Department of Anesthesiology, Chi Mei Medical Center, Liouying, Tainan City, Taiwan

**Keywords:** iron deficiency anemia, tinnitus, nutrition, cohort study, hazard ratio

## Abstract

**Background:**

Iron deficiency anemia (IDA) may compromise auditory function through cochlear hypoxia and altered hemodynamics; however, longitudinal evidence linking IDA to tinnitus remains limited. This study investigated the association between IDA and new-onset tinnitus in female patients using a large-scale cohort design.

**Methods:**

This retrospective cohort study utilized the TriNetX electronic health records network (2010–2022) to identify female patients with and without IDA, focusing exclusively on women to minimize occupational noise exposure confounding. We defined IDA as hemoglobin below 12 g/dL and ferritin below 30 ng/mL recorded within 3 months, while controls had levels above these thresholds. The primary outcome was new-onset tinnitus, and the secondary outcome was pulsatile tinnitus, both assessed at 1 and 3 years after the index date. After employing propensity score matching to balance baseline characteristics, we calculated the hazard ratio (HR) using Cox proportional hazards models and performed pre-specified subgroup analyses examining dose-response relationships by hemoglobin severity and age-stratified effects.

**Results:**

IDA was associated with a significantly higher risk of tinnitus at 1 year (HR 3.78, 95% confidence interval [CI]: 2.60–5.50; incidence 15.7 vs. 4.2 per 10,000 person-years) and at 3 years (HR 2.52, 95% CI 2.11–3.02; 18.6 vs. 7.4 per 10,000 person-years). Pulsatile tinnitus risk was similarly elevated at 3 years (HR 2.25, 95% CI 1.42–3.57). A clear dose-response relationship emerged, with severe IDA (hemoglobin < 10 g/dL) conferring highest 1-year risk (HR 5.74, 95% CI 3.24–10.16). Age-stratified analysis revealed differential vulnerability: older women (> 45 years) showed greater susceptibility to general tinnitus (HR 4.55 vs. 3.26), while younger women demonstrated exclusive risk for pulsatile tinnitus.

**Conclusion:**

IDA showed a significant dose-dependent association with new-onset tinnitus in women. These findings support routine IDA screening in women presenting with tinnitus and suggest that timely iron repletion may help reduce the risk of potentially preventable auditory dysfunction.

## 1 Introduction

Tinnitus, the perception of sound without an external acoustic stimulus, affects approximately 10–15% of the global population and significantly impairs quality of life through sleep disturbance, concentration difficulties, and psychological distress (1–4). Although multiple etiologies contribute to tinnitus development (5–7), systemic diseases that affect cochlear perfusion and oxygen delivery have emerged as important risk factors (8). Iron deficiency anemia (IDA), characterized by both depleted iron stores and reduced hemoglobin levels, represents a unique pathophysiological state that simultaneously compromises oxygen-carrying capacity and cellular iron-dependent processes (9, 10). The dual impact of IDA, combining anemia-related tissue hypoxia with iron depletion at the cellular level, may particularly affect the metabolically demanding auditory system (11, 12).

IDA remains the most prevalent form of anemia worldwide, disproportionately affecting women of reproductive age owing to menstrual losses and increased demands during pregnancy (13–15). Unlike isolated iron deficiency without anemia, IDA produces measurable reductions in oxygen delivery to peripheral tissues, including the cochlea. The cochlear structures, particularly the stria vascularis and organ of Corti, demonstrate exceptional vulnerability to hypoxic injury owing to their high metabolic requirements and limited collateral circulation (16, 17). Additionally, the anemic state of IDA triggers compensatory cardiovascular responses, including increased cardiac output and altered blood flow dynamics, which may manifest as pulsatile tinnitus (18). These hemodynamic changes, combined with reduced oxygen tension in the inner ear, may create a multifactorial mechanism through which IDA can precipitate both subjective and objective forms of tinnitus.

Despite cumulative evidence linking IDA to hearing loss (11, 19, 20), current literature focusing specifically on IDA and tinnitus remains scarce. Maihoub et al. demonstrated that lower hemoglobin significantly predicted higher tinnitus severity and bilateral tinnitus occurrence, highlighting a potential hematologic contribution to tinnitus pathophysiology (21). Additionally, Tang et al. (22) found that lower dietary intakes of iron were significantly associated with increased 10-year risk of incident tinnitus, supporting a nutritional link to tinnitus development. However, previous investigations have not adequately distinguished between the effects of IDA and other forms of anemia, nor have they examined dose-response relationships based on hemoglobin levels. Therefore, this study aimed to investigate the longitudinal association between IDA and new-onset tinnitus in female patients using a large-scale cohort design with propensity score matching, hypothesizing that IDA would be associated with a significant and potentially dose-dependent increase in the risk of tinnitus development.

## 2 Materials and methods

### 2.1 Data source

This retrospective cohort study utilized the TriNetX Analytics Network Platform, a federated health research network containing de-identified electronic health records from over 120 healthcare organizations across multiple countries. The database encompasses comprehensive clinical variables including demographics, diagnoses coded using the International Classification of Diseases (ICD-10-CM), procedures documented with Current Procedural Terminology (CPT) codes, laboratory measurements, and medication records. The TriNetX platform has been widely employed in numerous peer-reviewed clinical studies across various medical disciplines (23–25), demonstrating its reliability and validity as a research database. The Institutional Review Board of Chi Mei Medical Center approved this study (IRB number: 11403-E01) and waived the requirement for informed consent given the retrospective nature and use of de-identified data.

### 2.2 Study population

We identified female patients with and without IDA between January 1, 2010, and December 31, 2022, from the TriNetX database. The study focused exclusively on female patients because this population experiences higher rates of IDA due to menstrual blood loss and pregnancy-related demands while simultaneously having lower occupational noise exposure than males, thereby minimizing this important confounding factor for hearing-related outcomes. IDA was defined as concurrent hemoglobin levels below 12 g/dL and serum ferritin concentrations below 30 ng/mL (26), both recorded within a 3-month period to ensure temporal consistency between these diagnostic markers (index date). The control group comprised female patients without IDA, defined as those with hemoglobin levels above 12 g/dL combined with serum ferritin concentrations exceeding 30 ng/mL within the same 3-month timeframe. To address the potential bias from differential healthcare utilization patterns, we required all patients in the control group to have at least six hemoglobin measurements recorded during the study period, ensuring comparable levels of medical surveillance between groups.

### 2.3 Exclusion criteria

To focus on new-onset tinnitus and minimize confounding, we excluded patients with any prior diagnosis of tinnitus (ICD-10-CM H93.1) or pulsatile tinnitus (ICD-10-CM H93.A), sickle cell disorders (which independently affect cochlear function through vaso-occlusive mechanisms) (ICD-10-CM D57) (27), surgical procedures involving the inner or middle ear, neoplasms (given their potential for ototoxic treatments) (ICD-10-CM C00–D49), and advanced chronic kidney disease (stages 4–5 and end-stage renal disease) (ICD-10-CM N18.4–18.6), as uremic toxins and electrolyte imbalances may directly affect auditory function (28). Furthermore, patients in both cohorts were excluded if they had a history of ototoxic medication use (i.e., aminoglycosides, loop diuretics, or cisplatin) (29) or if they developed alternative forms of anemia (nutritional, hemolytic, aplastic, or unspecified anemia) during the follow-up period.

### 2.4 Data collection and propensity score matching

Propensity score matching was employed to balance baseline characteristics between the IDA and control groups, thereby minimizing confounding by indication. Baseline characteristics were extracted from the 3-year period preceding the index date to capture comprehensive patient profiles. The matching variables included demographic factors (age and race), body mass index, and comorbidities, including hypertension, heart failure, ischemic heart disease, diabetes mellitus, thyroid disorders, sleep disorders, liver diseases, cerebrovascular diseases, and systemic connective tissue disorders. Laboratory parameters incorporated into the matching algorithm included hemoglobin A1c values (reflecting glycemic control), serum albumin concentrations (indicating nutritional and inflammatory status), and estimated glomerular filtration rate (assessing renal function). The matching employed a greedy nearest-neighbor algorithm without replacement, using a caliper width of 0.1 standard deviations of the logit of the propensity score to ensure close matches while maintaining adequate sample size.

### 2.5 Outcomes

The primary outcome was the risk of new-onset tinnitus (ICD-10-CM H93.1) within 3 years of the index date, while the secondary outcome examined new-onset pulsatile tinnitus (ICD-10-CM H93.A), a distinct clinical entity that potentially reflects vascular etiology. A maximum follow-up of 3 years was chosen to balance sufficient observation time with data completeness, as longer follow-up substantially reduced patient retention within the TriNetX dataset and increased misclassification risk from changing anemia status or healthcare organization coverage. To provide insights into the temporal relationship between IDA and tinnitus development, we also evaluated outcomes at 1 year post-index date. A 1-month washout period was implemented, excluding patients who developed tinnitus within 1 month after the index date, to minimize detection bias from increased medical attention immediately following IDA diagnosis and to ensure adequate temporal separation between exposure and outcome.

### 2.6 Subgroup analyses and dose-dependent response

Subgroup analyses were conducted to explore effect modification and dose-response relationships, with independent propensity score matching performed within each subgroup to ensure optimal covariate balance for stratum-specific comparisons. First, we stratified patients by age (18–45 years versus over 45 years) to examine whether the association varied between younger adults (where IDA predominantly reflects menstrual losses) and older adults (where multiple comorbidities might influence both iron status and hearing). Second, to investigate a potential dose-dependent relationship, we categorized patients with IDA by hemoglobin severity: moderate IDA (hemoglobin 10–12 g/dL) versus severe IDA (hemoglobin < 10 g/dL), hypothesizing that more severe anemia would demonstrate stronger associations with tinnitus development due to greater tissue hypoxia and metabolic dysfunction.

### 2.7 Statistical analysis

Baseline characteristics were described using means with standard deviations for continuous variables and frequencies with percentages for categorical variables. Balance after matching was evaluated using standardized mean differences, with values under 0.1 indicating adequate balance between groups. Propensity score density functions were examined to ensure sufficient overlap between the groups. Event timing and cumulative incidence were explored using Kaplan-Meier estimates, with group differences assessed using log-rank tests. Hazard ratios (HR) with 95% confidence intervals (CI) were derived from Cox proportional hazards models, with the proportional hazards assumption verified using Schoenfeld residuals.

All analyses adhered to an intention-to-treat approach, retaining participants in their original groups regardless of subsequent changes in anemia status during follow-up, thereby preserving the benefits of randomization-like allocation achieved through propensity score matching. Missing data for baseline covariates and laboratory variables were handled by complete-case analysis within each healthcare organization contributing to the TriNetX network. Because TriNetX aggregates de-identified and quality-checked electronic health records, direct imputation was not feasible, but each participating institution provides data with validated completeness and quality-control standards, minimizing missingness in the analytic dataset. Statistical significance was defined as a two-sided *p* < 0.05. All analyses were conducted within the TriNetX platform using integrated statistical tools, ensuring consistency and reproducibility of the results.

## 3 Result

### 3.1 Patient selection and baseline characteristics before and after matching

**FIGURE 1:**
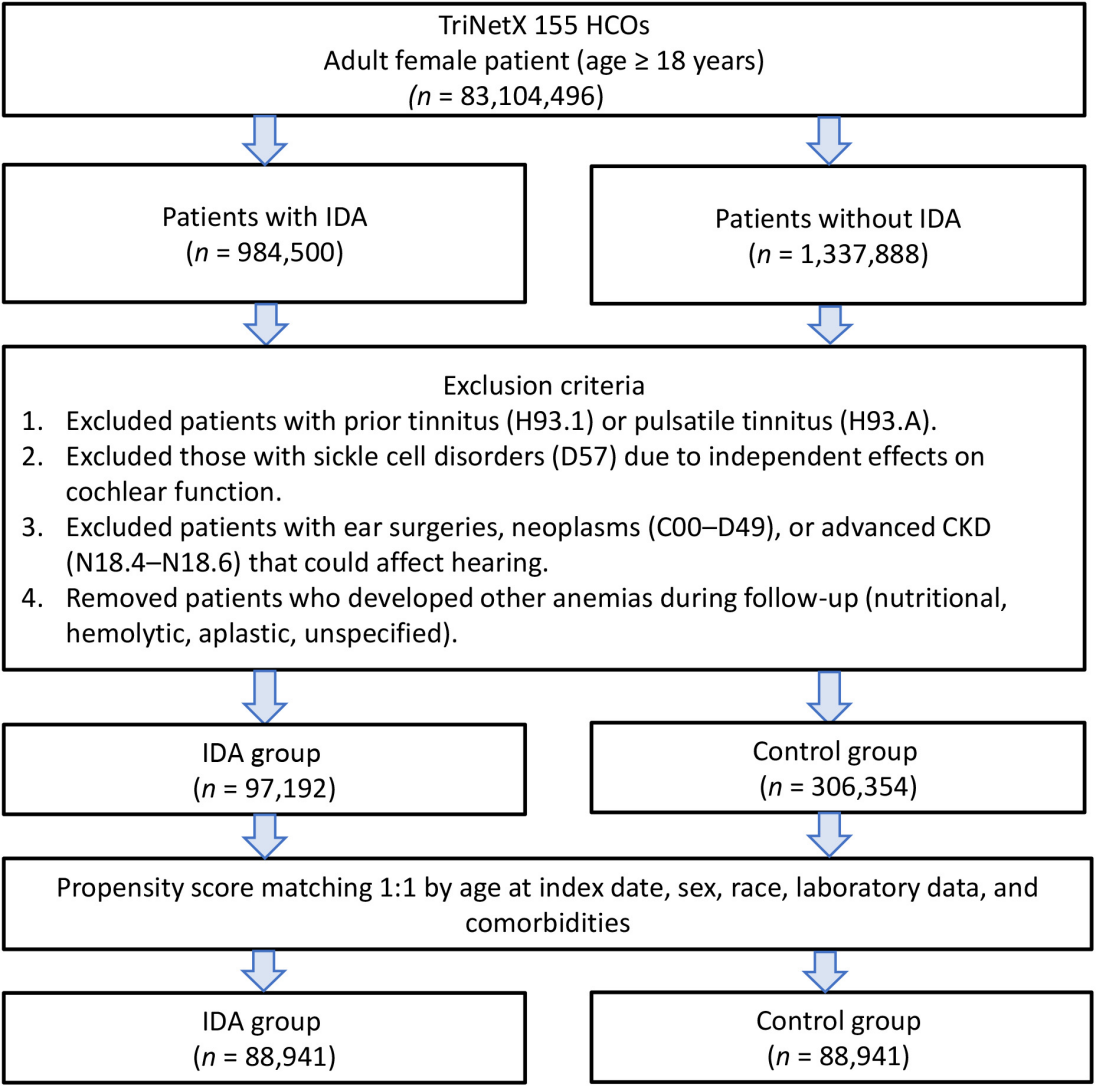
Patient selection flowchart from the TriNetX database. The flowchart illustrates the exclusion process applied to identify eligible patients with or without iron deficiency anemia (IDA).

**FIGURE 2:**
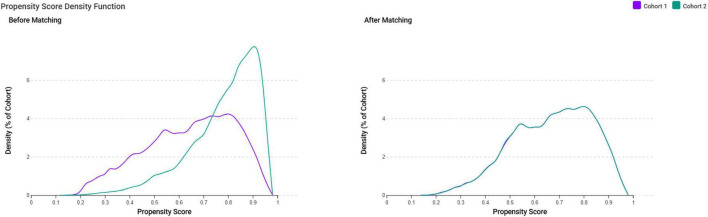
Propensity score density distributions before and after matching. The density plots demonstrate the distribution of propensity scores for the iron deficiency anemia group (Cohort 1, purple) and control group (Cohort 2, teal) before and after propensity score matching. Before matching, the two cohorts showed poor overlap in propensity score distributions, indicating substantial baseline differences. After matching, the distributions demonstrate excellent overlap, confirming successful balancing of baseline characteristics between groups and minimizing selection bias for subsequent outcome analyses.

After applying the exclusion criteria to eliminate pre-existing tinnitus and potential confounders, we identified 97,192 female patients with IDA and 306,354 controls without IDA. After propensity score matching, there were 88,941 patients in each group (Figure 1). The propensity score density function demonstrated excellent overlap between the matched groups (Figure 2), validating the matching approach and minimizing residual confounding.

After matching, all baseline characteristics achieved excellent balance, with standardized mean differences below 0.1, the threshold indicating negligible between-group differences (Table 1). The matched cohorts showed nearly identical demographic profiles, with mean ages of 39.9 years in both groups and comparable distributions across all racial categories. Important comorbidities that could influence tinnitus risk were also well balanced, including hypertension (15.0% vs. 16.2%), diabetes mellitus (7.2% vs. 7.5%), and thyroid disorders (9.7 vs. 10.3%). Both groups showed similar laboratory profiles, including normal albumin levels (45.5% vs. 48.2%) and adequate renal function (estimated Glomerular Filtration Rate > 60 mL/min/1.73 m^2^: 50.8% vs. 51.1%).

**TABLE 1:** Baseline characteristics of female patients with and without iron deficiency anemia before and after propensity score matching.

Variables	Before matching		After matching			
	IDA group (*n* = 97,192)	Control group (*n* = 306,354)	SMD^†^	IDA group (*n* = 88,941)	Control group (*n* = 88,941)	SMD†
**Patient characteristics**						
Age at index (years)	38.5 ± 16.9	49.9 ± 19.8	0.618	39.9 ± 16.8	39.9 ± 17.6	< 0.001
BMI ≥ 30 kg/m^2^	23,832 (24.5%)	65,467 (21.4%)	0.075	22,133 (24.9%)	23,719 (26.7%)	0.041
White	43,681 (44.9%)	144,802 (47.3%)	0.047	42,825 (48.2%)	44,931 (50.5%)	0.047
Unknown race	18,043 (18.6%)	109,987 (35.9%)	0.397	18,042 (20.3%)	17,166 (19.3%)	0.025
Black or African American	22,578 (23.2%)	30,842 (10.1%)	0.359	17,218 (19.4%)	16,038 (18.0%)	0.034
Other race	6,681 (6.9%)	10,842 (3.5%)	0.151	5,629 (6.3%)	5,645 (6.3%)	0.001
Asian	5,137 (5.3%)	8,357 (2.7%)	0.131	4,353 (4.9%)	4,335 (4.9%)	0.001
Factors influencing health status and contact with health services	54,826 (56.4%)	130,434 (42.6%)	0.279	48,723 (54.8%)	52,125 (58.6%)	0.077
**Comorbidities**						
Essential (primary) hypertension	13,856 (14.3%)	48,992 (16.0%)	0.048	13,304 (15.0%)	14,365 (16.2%)	0.033
Overweight and obesity	14,173 (14.6%)	37,380 (12.2%)	0.070	13,154 (14.8%)	14,192 (16.0%)	0.032
Dyslipidemia	9,774 (10.1%)	37,650 (12.3%)	0.071	9,514 (10.7%)	10,146 (11.4%)	0.023
Disorders of thyroid gland	9,043 (9.3%)	27,263 (8.9%)	0.014	8,584 (9.7%)	9,197 (10.3%)	0.023
Sleep disorders	6,682 (6.9%)	22,623 (7.4%)	0.020	6,435 (7.2%)	6,896 (7.8%)	0.020
Diabetes mellitus	6,615 (6.8%)	22,358 (7.3%)	0.019	6,386 (7.2%)	6,708 (7.5%)	0.014
Nicotine dependence	4,229 (4.4%)	14,233 (4.6%)	0.014	4,069 (4.6%)	4,108 (4.6%)	0.002
Diseases of liver	2,214 (2.3%)	14,071 (4.6%)	0.127	2,209 (2.5%)	2,500 (2.8%)	0.020
COVID-19	2,229 (2.3%)	13,731 (4.5%)	0.121	2,209 (2.5%)	2,475 (2.8%)	0.019
Cerebrovascular diseases	1,707 (1.8%)	8,873 (2.9%)	0.076	1,682 (1.9%)	1,753 (2.0%)	0.006
Systemic connective tissue disorders	1,399 (1.4%)	6,522 (2.1%)	0.052	1,382 (1.6%)	1,508 (1.7%)	0.011
Chronic kidney disease (CKD)	1,124 (1.2%)	6,408 (2.1%)	0.074	1,116 (1.3%)	1,234 (1.4%)	0.012
Alcohol related disorders	1,061 (1.1%)	4,454 (1.5%)	0.032	1,036 (1.2%)	1,077 (1.2%)	0.004
Malnutrition	805 (0.8%)	3,733 (1.2%)	0.039	792 (0.9%)	842 (0.9%)	0.006
Unspecified hearing loss	669 (0.7%)	2,359 (0.8%)	0.010	612 (0.7%)	652 (0.7%)	0.005
Conductive/sensorineural hearing loss	437 (0.5%)	1,457 (0.5%)	0.004	405 (0.5%)	415 (0.5%)	0.002
Hyperparathyroidism and other disorders of parathyroid gland	249 (0.3%)	818 (0.3%)	0.002	240 (0.3%)	256 (0.3%)	0.003
**Laboratory data**						
Hemoglobin A1c ≥ 9%	3,109 (3.2%)	15,516 (5.1%)	0.094	3,066 (3.4%)	3,137 (3.5%)	0.004
Albumin g/dL (≥ 3.5 g/dL)	41,289 (42.5%)	168,578 (55.0%)	0.253	40,455 (45.5%)	42,848 (48.2%)	0.054
eGFR > 60 mL/min/1.73 m^2^	45,887 (47.2%)	198,610 (64.8%)	0.361	45,215 (50.8%)	45,411 (51.1%)	0.004

IDA, iron deficiency anemia; BMI: body mass index; SMD, standardized mean differences; eGFR, estimated Glomerular Filtration Rate.

† SMD values < 0.1 indicate adequate balance between groups;

### 3.2 Association between IDA and risk of new-onset tinnitus

The primary analysis revealed a strong and clinically significant association between IDA and the development of new-onset tinnitus (Table 2). At 1-year follow-up, the occurrence of tinnitus was approximately four times more frequent among patients with IDA than among those without IDA (HR 3.78, 95% CI 2.60–5.50, *p* < 0.001), with an incidence of 15.7 versus 4.2 per 10,000 person-years. This substantial risk elevation suggests that IDA has relatively rapid effects on auditory function.

**TABLE 2:** Association between iron deficiency anemia and new-onset tinnitus at 1-year and 3-year follow-up.

Outcomes	IDA group (*n* = 88,941)		Control group (*n* = 88,941)		HR (95% CI)	*p*-value
	Events (n)	Incidence 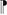	Events (n)	Incidence 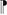		
**1-year outcome**						
Tinnitus	124	15.7	35	4.2	3.78 (2.60–5.50)	< 0.001
Pulsatile tinnitus^†^	–	–	–	–	–	–
**3-year outcomes**						
Tinnitus	391	18.6	174	7.4	2.52 (2.11–3.02)	< 0.001
Pulsatile tinnitus	54	2.6	27	1.2	2.25 (1.42–3.57)	< 0.001

IDA, iron deficiency anemia; HR, hazard ratio; CI, confidence interval; 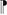
per 10,000 person-years.

† The patient count is too small, so detailed results cannot be displayed.

At the 3-year follow-up, the association remained pronounced but showed mild attenuation (HR 2.52, 95% CI 2.11–3.02, *p* < 0.001), with incidence rates of 18.6 versus 7.4 per 10,000 person-years (Table 2). The sustained yet slightly weakened association could reflect partial iron restoration in some individuals or adaptive physiological responses. Furthermore, IDA was associated with a significantly increased risk of pulsatile tinnitus at 3 years (HR 2.25, 95% CI 1.42–3.57, *p* < 0.001), a distinct entity frequently related to vascular pathology.

### 3.3 Dose-dependent analysis based on hemoglobin level

The dose-response analysis provided evidence of a biological gradient between anemia severity and tinnitus risk (Table 3). Among patients with moderate IDA (hemoglobin 10–12 g/dL, *n* = 76,479 for each group), the 1-year tinnitus risk was elevated 3.55-fold (95% CI 2.42–5.20, *p* < 0.001). Strikingly, patients with severe IDA (hemoglobin < 10 g/dL, *n* = 50,658 for each group) showed an even greater risk elevation at 1 year (HR 5.74, 95% CI 3.24–10.16, *p* < 0.001), demonstrating a clear dose-response relationship where more severe anemia conferred proportionally higher risk.

**TABLE 3:** Dose-dependent association between iron deficiency anemia severity and new-onset tinnitus based on hemoglobin levels.

Outcomes	Hb 10–12 g/dL (*n* = 76,479 for each group)		Hb < 10 g/dL (*n* = 50,658 for each group)	
	HR (95% CI)	*p*-values	HR (95% CI)	*p*-values
**1-year outcome**						
Tinnitus	3.55 (2.42–5.20)	< 0.001	5.74 (3.24–10.16)	< 0.001
Pulsatile tinnitus^†^	–	–	–	–
**3-year outcomes**						
Tinnitus	2.53 (2.11–3.03)	< 0.001	2.45 (1.94–3.09)	< 0.001
Pulsatile tinnitus	2.37 (1.53–3.68)	< 0.001	2.23 (1.24–4.00)	0.006

Hb, hemoglobin; HR, hazard ratio; CI, confidence interval.

† The patient count is too small, so detailed results cannot be displayed.

Interestingly, at 3-year follow-up, both moderate and severe IDA groups showed similar HRs for tinnitus (HR 2.53 and 2.45, respectively), suggesting potential convergence of risk over time. This pattern might indicate that while severe anemia causes immediate auditory dysfunction, chronic mild-to-moderate iron deficiency may ultimately produce comparable cumulative damage. For pulsatile tinnitus at the 3-year follow-up, the risk was similar in patients with moderate and severe IDA.

### 3.4 Subgroup analysis based on age

Age-stratified analyses revealed important effect modifications, with older women showing greater vulnerability to IDA-associated tinnitus (Table 4). Women over 45 years of age with IDA experienced a 4.55-fold increased risk of tinnitus at 1 year, compared to a 3.26-fold increase in younger women. This age-related susceptibility persisted at 3 years (HR: 3.03 vs. 2.52 for older and younger women). Conversely, pulsatile tinnitus showed an interesting age-dependent pattern, with significant risk elevation only in younger women at 3 years (HR 2.86, 95% CI 1.50–5.44, *p* < 0.001), while older women showed no significant association (HR 1.44, 95% CI 0.74–2.82, *p* = 0.281). This divergent pattern suggests that vascular adaptations to IDA may differ across age groups, with younger women potentially experiencing more pronounced hemodynamic changes that manifest as pulsatile tinnitus.

**TABLE 4:** Age-stratified analysis of the association between iron deficiency anemia and new-onset tinnitus.

Outcomes	18–45 years (*n* = 42,976 for each group)		> 45 years (*n* = 42,189 for each group)	
	HR (95% CI)	*p*-values	HR (95% CI)	*p*-values
**1-year outcome**						
Tinnitus	3.26 (1.91–5.56)	< 0.001	4.55 (2.55–8.14)	< 0.001
Pulsatile tinnitus^†^	–	–	–	–
**3-year outcomes**						
Tinnitus	2.52 (1.93–3.29)	< 0.001	3.03 (2.34–3.92)	< 0.001
Pulsatile tinnitus	2.86 (1.50–5.44)	< 0.001	1.44 (0.74–2.82)	0.281

HR, hazard ratio; CI, confidence interval.

† The patient count is too small, so detailed results cannot be displayed.

## 4 Discussion

This large-scale cohort study identified an association between IDA and new-onset tinnitus in female patients. Women with IDA exhibited a nearly four-fold increased risk of developing tinnitus at 1 year and a sustained 2.5-fold elevation at 3 years compared to matched controls. The association displayed a clear dose-response gradient, with severe IDA (hemoglobin < 10 g/dL) conferring the highest risk at 1 year (HR 5.74). Analyses stratified by age demonstrated differing susceptibility profiles, with older women showing heightened vulnerability to subjective tinnitus and younger women displaying a greater risk for pulsatile tinnitus.

The pathophysiology connecting IDA to tinnitus may involve multiple interconnected mechanisms. First, reduced hemoglobin levels compromise oxygen delivery to the metabolically demanding cochlea, particularly affecting the stria vascularis, which maintains the endocochlear potential essential for auditory transduction (16, 17). Disruption of stria vascularis function can lead to impaired potassium recycling, reduced endocochlear potential, and secondary outer hair-cell dysfunction (30). Iron deficiency further compromises hair-cell metabolism by impairing mitochondrial oxidative phosphorylation and increasing reactive oxygen species generation, both of which promote cochlear energy failure and synaptic damage (31). In parallel, chronic auditory deafferentation and hypoxia-induced neural stress may enhance central gain within auditory cortical and brainstem pathways, producing persistent tinnitus perception even after peripheral recovery (32). Second, iron deficiency disrupts mitochondrial function and cellular energy metabolism because iron serves as a critical cofactor for cytochrome enzymes and oxidative phosphorylation (33). Cochlear hair cells, owing to their high energy demands for mechanotransduction and synaptic signaling, are especially susceptible to metabolic compromise (31). Third, IDA triggers compensatory cardiovascular responses, including increased cardiac output, altered blood viscosity, and turbulent flow patterns that may manifest as pulsatile tinnitus. Fourth, iron deficiency affects neurotransmitter synthesis, particularly the dopamine and serotonin pathways involved in auditory processing and tinnitus perception (34). Finally, chronic tissue hypoxia may induce aberrant neural plasticity and spontaneous neural activity in the auditory pathway (35).

The temporal pattern observed, with a peak effect size at 1 year followed by attenuation at 3 years, reveals important physiological insights. The early elevated risk suggests that acute metabolic stress from IDA rapidly compromises cochlear function, potentially triggering immediate compensatory mechanisms, including increased spontaneous firing rates in auditory neurons and maladaptive central gain adjustments. The decline in risk from HR 3.78 to 2.52 likely reflects several processes, including partial iron repletion via treatment or dietary improvement and physiological adaptation to chronic hypoxia through enhanced oxygen extraction efficiency. However, the persistent elevation at 3 years indicates that IDA induces lasting structural or functional alterations in the auditory system. This may include permanent damage to the cochlear microvasculature, persistent changes in central auditory processing pathways, or the establishment of aberrant neural circuits that maintain tinnitus perception despite hemoglobin normalization.

The observed dose–response pattern may support causal inference and indicates, though it does not confirm, that threshold effects may exist in auditory vulnerability. Severe IDA (hemoglobin < 10 g/dL) produced a markedly higher initial risk, suggesting that profound tissue hypoxia overwhelms compensatory mechanisms. This steep increase likely reflects the crossing of a critical threshold where oxygen delivery becomes insufficient to maintain normal cochlear metabolism, triggering cascading cellular dysfunction. Interestingly, the convergence of effect size at 3 years (2.53 vs. 2.45) suggests that while severe anemia causes more immediate damage, chronic mild-to-moderate deficiency produces comparable cumulative injury through sustained low-grade hypoxia and metabolic stress. This pattern emphasizes that both the severity and duration of IDA contribute to auditory pathology.

Age-related differences between subjective and pulsatile tinnitus indicate that these two forms likely involve separate pathophysiological processes. The increased vulnerability of older women to subjective tinnitus (HR 4.55 vs. 3.26) is probably attributable to age-associated changes. Reduced cochlear reserve, cumulative oxidative stress, and microvascular aging may further heighten susceptibility through impaired hair-cell metabolism and weakened antioxidant defense, consistent with presbycusis-related changes (36). Conversely, the higher pulsatile tinnitus risk among younger women may reflect greater cardiovascular reactivity to anemia, manifested by increased cardiac output and vascular turbulence, producing audible vascular sounds even in the absence of direct cochlear injury. The absence of pulsatile tinnitus risk in older women may reflect age-related vascular stiffening that dampens flow-related sounds or reduces cardiovascular reserve, limiting hemodynamic compensation. This age-dependent physiological distinction suggests that IDA influences tinnitus through different predominant pathways across life stages. Importantly, the pathophysiology of pulsatile tinnitus should be distinguished from that of subjective tinnitus. While the latter primarily reflects cochlear hypoxia or neural hyperactivity, pulsatile tinnitus often arises from systemic hemodynamic alterations. Reduced blood viscosity, increased cardiac output, and turbulent arterial flow may generate vascular sounds perceived as pulsatile tinnitus (37), even in the absence of cochlear injury. This distinction underscores that IDA-related pulsatile tinnitus is likely mediated by circulatory rather than intrinsic auditory mechanisms.

Regarding the clinical implications, the strong association observed supports the importance of considering IDA in the evaluation of tinnitus, particularly in women. This dose-dependent pattern suggests that even mild IDA may contribute to increased tinnitus risk, although causal inference cannot be established. The temporal pattern observed within the first year suggests that timely iron correction may help reduce auditory risk, but further studies are needed to confirm this possibility. From a clinical audiology perspective, patients with severe IDA may benefit from baseline and follow-up auditory evaluations, including pure-tone audiometry, otoacoustic emissions, and auditory brainstem response testing to detect subclinical hearing dysfunction. Incorporating validated tinnitus handicap instruments could also help quantify symptom burden and treatment response. Moreover, assessing IDA should be considered in the differential diagnosis of idiopathic tinnitus to ensure timely recognition of hematologic contributors. In younger women with pulsatile tinnitus, IDA should be considered in the differential diagnosis along with vascular abnormalities. In current study, although the relative risk increase was substantial, the absolute incidence of newly diagnosed tinnitus in both cohorts was low. This likely reflects underdiagnosis in real-world data, where many patients with mild or transient tinnitus do not seek medical attention.

This study had several limitations that merit consideration. First, while our findings provide strong evidence of an association between IDA and tinnitus, they do not establish causality. Residual confounding from unmeasured factors such as occupational or recreational noise exposure, psychological stress, nutritional patterns, or other lifestyle variables may still account for part or all of the observed associations. Therefore, our conclusions should be interpreted as suggestive rather than definitive. Second, we lacked data on iron supplementation compliance, dietary iron intake, and treatment response, which could influence the outcomes. Third, audiometric or auditory brainstem response data were not available within the TriNetX platform, precluding direct assessment of hearing thresholds. Consequently, we could not determine whether tinnitus in our cohort developed independently of measurable hearing loss. In addition, tinnitus severity, characteristics, and impact on quality of life were not captured by diagnostic codes alone. Fourth, noise exposure history and genetic predisposition to hearing disorders were unavailable for adjustment. Fifth, IDA exposure in this study was defined at a single baseline time point and did not capture subsequent changes in anemia status. Some patients may have received iron therapy or nutritional correction after the index date, which could attenuate the long-term risk estimates. Therefore, our findings reflect associations with baseline IDA rather than persistent or recurrent anemia over time. In addition, we acknowledge that the requirement of at least six hemoglobin measurements in the control group, although intended to ensure comparable medical surveillance, may limit generalizability by selecting individuals with more frequent healthcare utilization. This criterion could therefore introduce a degree of selection bias, which should be considered when interpreting our findings. Finally, the female-only cohort limits generalizability to males, although this design choice strengthened internal validity by minimizing occupational noise confounding. In addition, no audiologist was directly involved in this study, which may limit audiological interpretation despite multidisciplinary review.

## 5 Conclusion

This large cohort study identified an association between IDA and new-onset tinnitus in women, with a 3.78-fold increased risk at 1 year and sustained 2.52-fold elevation at 3 years, alongside a significant association with pulsatile tinnitus. These findings support the consideration of IDA screening in women with tinnitus and suggest that timely iron repletion may help reduce the risk of potentially preventable auditory dysfunction, although causality cannot be confirmed. Future research should investigate whether iron supplementation reduces tinnitus incidence, explore biomarkers predicting individual susceptibility, and examine long-term auditory outcomes following IDA treatment to optimize preventive strategies for this common and debilitating condition.

## Data Availability

The raw data supporting the conclusions of this article will be made available by the authors, without undue reservation.
